# Mitral Valve Abnormalities as Predictors of Procedural Success in Alcohol Septal Ablation: A Pilot Study

**DOI:** 10.3390/jcm15031031

**Published:** 2026-01-28

**Authors:** Raluca Coifan, Monica Mircea, Alexandru Silvius Pescariu, Oana Voinescu, Bogdan Enache, Laurentiu Pascalau, Mihai-Andrei Lazăr, Ionut Golet, Adrian Sturza, Constantin Tudor Luca, Adina Ionac, Cristian Mornos

**Affiliations:** 1 Department VI—Cardiology, “Victor Babes” University of Medicine and Pharmacy of Timisoara, E. Murgu Sq. No. 2, 300041 Timisoara, Romania; sosdean.raluca@umft.ro (R.C.); mornos.cristian@umft.ro (C.M.); 2 Research Centre of the Institute of Cardiovascular Diseases Timisoara, G. Adam Str. No. 13A, 300310 Timisoara, Romania; 3 Department of Management and Entrepreneurship, Faculty of Economics and Business Administration, West University of Timișoara, J. H Pestalozzi No. 16, 300115 Timisoara, Romania; 4 Department III—Pathophysiology, “Victor Babes” University of Medicine and Pharmacy of Timisoara, E. Murgu Sq. No. 2, 300041 Timisoara, Romania; 5 Centre for Translational Research and Systems Medicine, “Victor Babes” University of Medicine and Pharmacy of Timisoara, E. Murgu Sq. No. 2, 300041 Timisoara, Romania

**Keywords:** hypertrophic obstructive cardiomyopathy, alcohol septal ablation, mitral valve apparatus, mitral coaptation point, mitral leaflet projection, redundant anterior mitral leaflet length, pressure gradient reduction

## Abstract

**Background/Objectives**: Alcohol septal ablation (ASA) is an established interventional therapy for patients with obstructive hypertrophic cardiomyopathy (OHCM) who remain symptomatic despite optimal medical treatment. Nevertheless, 10–20% of patients fail to achieve a satisfactory hemodynamic or clinical response, highlighting the need for improved patient selection. Given that mitral valve (MV) morphology plays a central role in left ventricular outflow tract (LVOT) obstruction, we aimed to evaluate the impact of MV anatomical parameters on ASA outcomes. **Methods**: We retrospectively analyzed 38 OHCM patients who underwent ASA and had complete echocardiographic data before and at 6-month follow-up. Patients were stratified into responders (*n* = 32, defined as >50% reduction in LVOT pressure gradient and/or residual LVOT gradient < 50 mmHg) and non-responders (*n* = 6, <50% reduction or persistent gradient ≥ 50 mmHg), consistent with criteria used in previous ASA outcome studies. MV parameters—including redundant anterior mitral leaflet (AML) length, posterior mitral leaflet (PML) projection, and anterior displacement of the coaptation point (AML/PML projection ratio)—were compared between groups. **Results**: Non-responders demonstrated significantly greater AML redundancy (13.16 ± 1.72 vs. 9.96 ± 1.99 mm, *p* < 0.001), larger PML projection (18.5 ± 3.78 vs. 13.65 ± 3.8 mm, *p* = 0.006), and lower AML/PML projection ratio (0.80 ± 0.15 vs. 1.34 ± 0.45, *p* = 0.007). These parameters were associated with reduced post-procedural LVOT gradient reduction in univariate logistic regression (*p* = 0.01, *p* = 0.027, *p* = 0.015, respectively). Multivariate modeling was not pursued due to collinearity among MV parameters and the limited number of non-responder events, which precluded robust adjustment. **Conclusions**: Mitral valve morphological features—particularly redundant AML, greater PML projection, and anterior displacement of the coaptation point—were associated with suboptimal ASA outcomes in univariate analysis. These data emphasize the need for comprehensive MV imaging in pre-procedural assessment. Integrating MV morphology into current selection algorithms may refine ASA patient selection and improve long-term success rates.

## 1. Introduction

Hypertrophic cardiomyopathy (HCM) is one of the most frequent inherited cardiomyopathies, with a prevalence of 1:500–1:200 in the general population; however, only ~1 in 3000 individuals present with clinically manifest disease. The spectrum of complications includes progressive heart failure, atrial and ventricular arrhythmias, and sudden cardiac death (SCD) [1]. HCM is defined by unexplained left ventricular wall thickness ≥15 mm (or ≥13 mm in patients with positive family history or sarcomeric mutation), after exclusion of secondary causes of hypertrophy [1,2,3,4].

The phenotype may be variable from dominantly interventricular septum (IVS) and/or other LV wall forms to midventricular and apical forms. Dynamic LVOT obstruction occurs in approximately two-thirds of HCM patients and typically results from basal interventricular septal hypertrophy in combination with MV structural anomalies [1,4,5,6,7,8,9]. During systole, besides the significant narrowing of the LVOT secondary to the basal IVS hypertrophy, abnormal flow currents also form inside the geometrically modified LV, which, acting on a modified mitral valve, will induce the well-known systolic anterior motion (SAM), which further contributes to the obstruction [8,10,11]. Among the MV abnormalities, elongated and redundant anterior mitral leaflet (AML) and/or posterior mitral leaflet (PML), leaflet thickening, leaflet prolapse and/or restriction (especially PML), abnormal chordal insertion, and displaced or hypertrophied papillary muscles are common. In some cases, the papillary muscles insert directly onto the AML, bypassing the chordae tendineae. Because of its dimensions reported to the PML and MV annulus and of the anterior displacement of the coaptation point, a variable part of the AML length will not participate in the coaptation—the so-called redundant segment. Some studies demonstrated that 55% to 65% of cases associate elongated and redundant mitral valve leaflets, and leaflet thickening or prolapse is found in 70% of cases, while 60 to 75% of patients have abnormal chordal or papillary muscle insertion and anterior displacement of the mitral coaptation point [7,10,11]. Also anomalies of the mitral valve annulus consisting of a more acute aortomitral angle and a larger annulus height were described [10]. All these abnormalities can generate or exacerbate systolic anterior motion (SAM), which further narrows the LVOT and often causes secondary mitral regurgitation (MR) [8,11]. Notably, a significant anterior displacement of the mitral coaptation line and an excessive redundant AML segment seem to be often implicated in obstruction [10]. The role of MV morphology in LVOT obstruction severity is clinically relevant, as both primary structural abnormalities and secondary adaptive remodeling contribute to hemodynamic compromise. Septal reduction therapy is indicated in symptomatic hypertrophic obstructive cardiomyopathy (HOCM) patients refractory to medical therapy. According to the 2023 ESC Guidelines for the management of cardiomyopathies, surgical septal myectomy is considered the “gold standard” for gradient reduction with class I, level of evidence B indication [2,3]. It provides complete, long-lasting obstruction reduction, good survival in the long term, and it allows for simultaneous mitral valve and or papillary muscles correction when needed. However, in high-volume, specialized HOCM centers, alcohol septal ablation is indicated, class IIa, level of evidence B. It is a catheter-based, less invasive alternative for patients with suitable coronary anatomy for the procedure or high surgical risk. Both procedures are effective so the recommendations are made based on a multiparametric map [12,13,14,15]. Thus, pre-procedural imaging is critical to identify patients less likely to benefit from ASA [13,16], a procedure that has become more popular due to the fact that it relieves the patients from the surgical stress, provides faster recovery and has equivalent long-term benefits in appropriately selected patients [17,18,19,20]. ASA may be less successful in the presence of certain anatomic features, particularly when MV anomalies are prominent, as it exclusively targets the septal myocardium perfused by a suitable septal perforator artery [12,13,14,15]. Despite its high overall efficacy (>80% procedural success), a subset of patients (10–20%) exhibit persistent obstruction, often due to non-septal contributing factors [15]. We hypothesized that MV anatomical parameters may predict which patients are less likely to respond favorably and evaluated echocardiographic features in this context.

## 2. Materials and Methods

### 2.1. Study Design

We retrospectively included in this study a total of 38 consecutive patients with hypertrophic obstructive cardiomyopathy who underwent ASA in our hospital between 2018 and 2022, according to current guideline recommendations [2,3]. All the patients were thoroughly evaluated clinically and through transthoracic ultrasound before the procedure, as well as after the procedure at ≥6 months, when inflammation is likely to be resolved and the scar consolidated. The same device was used for evaluating each patient before and after, and the patients were evaluated by the same examiner in order to eliminate any bias and maintain consistency. The study protocol complied with the principles of the Declaration of Helsinki and received approval from the Ethics Committee of the Institute of Cardiovascular Diseases, Timisoara, Romania (approval no. 745/2 February 2023). Based on the obtained values, the patients were classified into the following groups.

### 2.2. Definition of Responders and Non-Responders

Based on definitions used in major studies [15,18,21], we classified the patients into 2 categories, which rely on hemodynamic endpoints as clinically meaningful surrogates of symptomatic improvement and prognosis. Patients were classified as responders if they demonstrated (i) a ≥50% reduction in LV outflow tract (LVOT) pressure gradient compared with baseline, and/or (ii) a residual resting or provoked LVOT gradient < 50 mmHg at 6 months. Non-responders were defined by <50% reduction and/or a persistent LVOT gradient ≥ 50 mmHg, or by early rebound obstruction.

### 2.3. ASA Procedure

ASA was performed under fluoroscopic and contrast echocardiographic guidance using SonoVue^®^ (Bracco Imaging, Milan, Italy). Only patients with suitable septal branch anatomy were considered eligible, defined by contrast localization to the basal anterior interventricular septum without spillover. Special care was taken in order to exclude contrast enhancement in other territories (suggestive of collaterals between the septal branch and other coronary branches) and to correctly establish the target septal branch(es) territory, which had to include the AML-IVS contact situs during the systolic anterior movement. Both the first and the second septal branches’ territories were evaluated by contrast echocardiography, considering a possible merge of their territories, if the target septal zone at the site of dynamic obstruction had an incomplete contrast opacification when evaluating the first septal artery. If suitable, the second septal artery was also targeted in these cases, in order to completely address the target septal myocardium and to preclude a suboptimal result due to an incomplete scar. Ethanol (approximately 2 mL) was slowly infused into the target septal branch(es), in order to induce the small, controlled area of necrosis in the basal interventricular septum.

### 2.4. Clinical Data

All patients had a thorough clinical evaluation (complete anamnesis and physical examination) before and after the procedure, 6 months follow-up included. Heart failure severity was classified according to the New York Heart Association (NYHA) functional classification. Data regarding comorbidities and heart failure evolution, as well as demographic data, was gathered from the patient files kept in the hospital’s electronic archive and introduced in the specially designed database for statistical analysis. Patients with incomplete data were excluded.

### 2.5. Echocardiography Protocol

All patients underwent comprehensive transthoracic echocardiography before ASA and at follow-up. The echocardiographic parameters were obtained by using a Vivid E 95 echo scanner (General Electric, Milwaukee, WI, USA). Offline image analysis was performed using both the internal software of the scanner and the ECHOPAC (version 203) external software. Standard measurements included LV wall thickness, LV diameters, chamber volumes, left atrial volume, LV ejection fraction (Simpson biplane), and LVOT gradients at rest and after Valsalva maneuver while also focusing on the morphology and movement of the mitral valve.

LV dimensions and function. LV wall thickness was assessed in parasternal short-axis views (basal, mid-ventricular, apical) and verified in parasternal long-axis views. Calipers were positioned at the endocardial and epicardial borders, avoiding left and/or right ventricle trabecular inclusion. LV diameters were measured in the parasternal long-axis view at the mitral leaflet tips. LV volumes and ejection fraction were calculated in apical four- and two-chamber views using the biplane Simpson method, and left atrial volume was measured similarly. Volumes were indexed to body surface area [1,22].

- LVOT gradients. The maximal LVOT pressure gradient was obtained by continuous-wave Doppler in apical five-chamber view, after confirming aliasing with pulsed-wave Doppler. For low post-procedural gradients, pulsed-wave Doppler was used. Care was taken to avoid contamination of LVOT signals with mitral regurgitation (MR), based on color Doppler flow mapping and analysis of Doppler envelope morphology (dagger-shaped late systolic peak for LVOT vs. parabolic, earlier-peaking high-velocity signal for MR) and also to exclude any fixed obstruction in the LVOT (i.e., subaortic stenosis) [10,23]. Maximal LVOT gradients (both before and after ASA) and the associated mitral regurgitation severity, used for statistical analysis, were evaluated under provocative maneuvers (Valsalva maneuver).

#### Mitral Valve Assessment

Dedicated mitral valve (MV) measurements were verified independently by two experienced echocardiographers, for reliability reasons. Analyses were conducted using standardized 2D transthoracic echocardiographic acquisitions, with particular attention to avoiding off-axis imaging.

**Figure 1:**
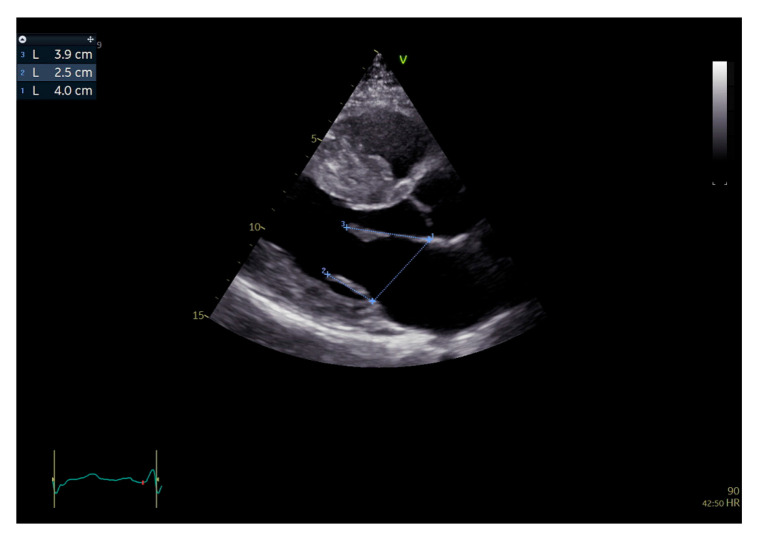
Two-dimensional transthoracic echocardiography, parasternal long axis view, end-diastolic frame. Measurement of the mitral valve annulus antero-posterior diameter (1) from the AML insertion point on the aortic root to the PML insertion point on the left atrial posterior wall and leaflets, from the posterior insertion point to the tip of the PML (2) and from the anterior insertion point to the tip of the AML (3).

**Figure 2:**
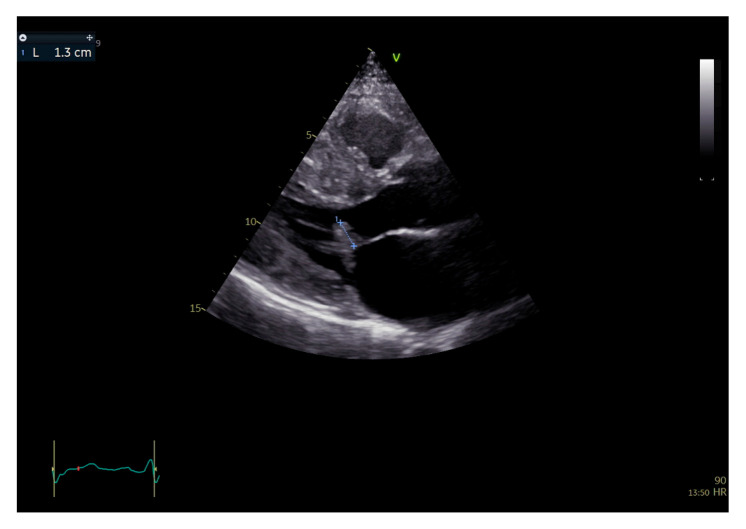
Two-dimensional transthoracic echocardiography, parasternal long axis view, end-systolic frame. Measurement of the redundant anterior mitral leaflet, the excessive tissue with systolic protrusion in the LVOT, from the mitral leaflets’ coaptation point to the tip of the AML.

**Figure 3:**
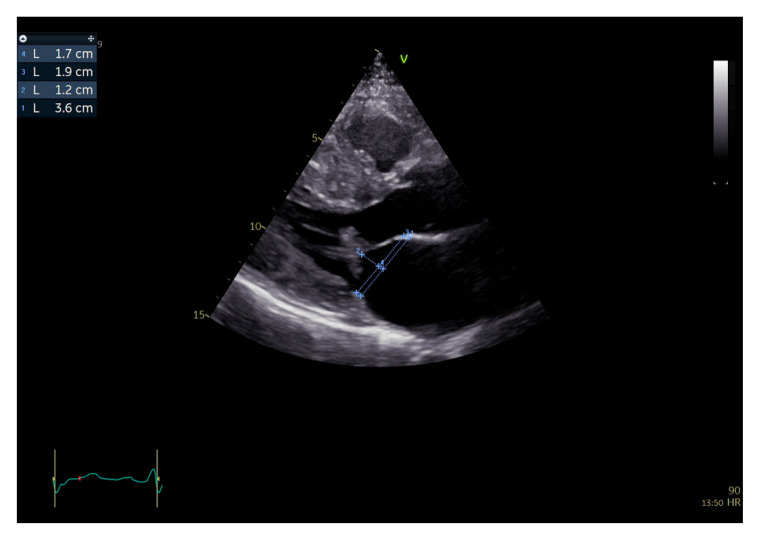
Two-dimensional transthoracic echocardiography, parasternal long axis view, end-systolic frame. Measurement of the mitral valve annulus antero-posterior diameter (1) between each leaflet insertion point, and mitral valve leaflets’ projection on the mitral valve annulus, as the distance between each insertion point and a line (2) drawn through the coaptation point, perpendicular to the annulus (AML projection—3, PML projection—4).

- Annulus and leaflet lengths. The MV annulus diameter was measured in parasternal long-axis view, at end-diastole and end-systole, from the anterior (junction point of the anterior mitral valve leaflet and the aortic root) to the posterior annulus (junction point of the posterior mitral valve leaflet and left atrial posterior wall). The anterior (AML) and posterior (PML) leaflets (A2 and P2 segments) were measured at end-diastole when maximally extended parallel to the LV wall (Figure 1), from the insertion point to the tip of the leaflet. The AML/PML length ratio was calculated [8,10,24].
- Redundant AML. Redundant AML length was defined as the excessive valvular tissue that extends beyond the coaptation point in systole, protruding in the LVOT-systolic anterior movement (SAM). It was measured in the parasternal long axis view, as the distance between the coaptation point and the leaflet tip at mid to late systole, at maximal systolic anterior motion (Figure 2) [10,23].
- Leaflet projections and coaptation point displacement. These variables were also measured in parasternal long-axis view at end-systole. In this regard, a perpendicular line to the annulus was drawn through the coaptation point (Figure 3). The projection length of each leaflet on the mitral annulus was measured from the annular insertion to this line. The AML/PML projection ratio was calculated by dividing the measured values to describe the degree of anterior displacement of the coaptation point [10]. A lower value of this ratio (a shorter AML projection and a longer PML projection) represents a more anterior displacement of the coaptation point.

All measurements were averaged over three cardiac cycles in sinus rhythm or five cycles in atrial fibrillation.

### 2.6. Statistical Analysis

Analyses were performed using Jamovi v.2.6.19. Continuous variables are expressed as mean ± SD, categorical variables as *n* (%). Between-group comparisons used Student’s *t*-test or Fisher’s exact test. Logistic regression was used to explore univariate associations between MV parameters and response status. Multivariate analysis was not performed due to collinearity between MV parameters and insufficient events-per-variable (6 non-responders), which would violate accepted statistical standards (≥10–15 events per variable) [25]. A *p* value of <0.05 was considered to be statistically significant.

## 3. Results

The 38 ASA patients included in this study were divided according to the success of the procedure in two groups. A total of 32 patients (84.21%) were classified as responders according to the echocardiographic results, while 6 patients (15.78%) were classified as non-responders.

### 3.1. Clinical Profiles

There were no statistically significant differences between responders and non-responders in terms of demographic data, baseline NYHA class, or comorbidities (Table 1). None of the included patients had significant coronary artery disease, severe primary valvular problems and/or other pathology necessitating cardiac surgery. All patients had symptomatic LVOT obstruction on maximum tolerated medical therapy at the moment of the intervention.

All diabetic patients had type 2 diabetes mellitus. Based on their glomerular filtration rate, two of the patients with chronic kidney disease in the responder group had stage G3a, one stage G3b and one stage G2, whereas the patient with chronic kidney disease in the non-responder group had stage G2. Regarding arterial hypertension, 11 patients had grade 2 and 12 patients had grade 3 in the responder group, whereas 2 patients had grade 2 and one had grade 3 in the non-responder group. Atrial fibrillation was paroxysmal in all 3 patients experiencing this arrhythmia. One patient had grade 2 obesity in the responder group, whereas the rest had grade 1.

There was a significant improvement in NYHA class in the responder group after the procedure (1.53 ± 0.5 vs. 2.68 ± 0.53, *p* < 0.0001), with 17 (53.12%) patients classified as NYHA II, and 15 (46.87%) patients as NYHA I but also in the non-responders’ group, even if weaker (1.83 ± 0.4 vs. 2.5 ± 0.54, *p* = 0.03), with 1 (16.66%) patient classified as NYHA I and the remaining 5 (83.33%) as NYHA II, meaning there was symptomatic improvement even with a suboptimal decrease in LVOT pressure gradient.

### 3.2. Echocardiographic Profiles

Baseline echocardiographic characteristics—including left ventricular volumes, ejection fraction, left atrial size, and resting/provoked LVOT gradients—were similar between the two groups (Table 2).

During the procedure, both the first and the second septal branches were established as target arteries by contrast echocardiography and obliterated in two patients (6.25%) from the responder group and two patients (33.33%) from the non-responder group. In all remaining patients, the first septal artery was targeted as it completely supplied the target myocardium.

At follow-up, the responder group showed a substantial reduction in provoked LVOT gradient (mean decrease of 77.97%, *p* < 0.001), whereas the non-responders had only a 35.14% decrease (*p* = 0.003), insufficient to alleviate hemodynamic obstruction. The difference reached statistical significance but was a less than 50% decrease as compared to baseline, with a hemodynamically significant residual obstruction. All patients in the non-responder group had a postprocedural hemodynamically significant obstruction, even without provocative maneuvers. In both groups there was a reduction in the basal IVS thickness, by 3.43 ± 2.72 mm in the responder group (19.96 ± 2.79 mm vs. 23.4 ± 2.98 mm) and by 2.83 ± 1.83 mm in the non-responder group (21.66 ± 3.01 mm vs. 24.5 ± 4.13 mm). The difference between the two groups did not reach statistical significance (*p* = 0.6).

### 3.3. Mitral Valve Echocardiographic Parameters

Key differentiating features between responders and non-responders included significantly longer redundant AML, larger PML projection, and lower AML/PML projection ratios (Table 3). These findings support the hypothesis that a more anteriorly displaced coaptation point and abnormal leaflet geometry may contribute to persistent obstruction.

All patients had mild mitral regurgitation 6 months after the procedure, except for a number of four patients in the responders’ group (12.5%) who had no to trivial mitral regurgitation. Six patients in the responders’ group still had a subtle residual mitral valve SAM (18.75%). In the non-responders’ group, all patients had residual SAM.

### 3.4. Impact of the Mitral Valve Echocardiographic Parameters on ASA Success

The redundant AML, PML projection and the anterior displacement of the coaptation point (AML projection/PML projection decrease) showed a statistically significant correlation with the percentage of LVOT PG decrease. An increased value of the redundant AML and PML projection, and a lower value of the AML projection/PML projection induce a higher percentage of LVOT PG decrease (*p* < 0.01, *p* < 0.05 and *p* < 0.05, respectively). A higher value of the IVS, LV anterior wall, AML, PML and a lower value of the AML projection had a weak correlation with the percentage of LVOT PG decrease but without reaching statistical significance (Table 4).

On the other hand, we found a statistically significant correlation between the thickness of the basal segments of the IVS and LV anterior wall, the AML and PML length, the redundant AML and the total length of the AML as well as the total length of the PML. In the same line, the projections of AML and PML correlated with the total length of the corresponding leaflet, and the AML/PML projection ratio decreased with the PML length (Table 4).

Increased length of redundant AML (OR = 0.47 (95% CI: 0.264–0.838)), increased PML projection (OR = 0.763 (95% CI: 0.601–0.969)), and reduced AML/PML projection ratio (OR = 130.096 (2.56–6611.615)) were associated with procedural success in univariate analysis (Table 5).

All non-responder patients had a subunitary AML projection/PML projection ratio (a value less than 1), whereas only three responder patients had that result.

## 4. Discussion

In predicting the outcomes of ASA, the present study underscores the pivotal role of mitral valve (MV) morphology. Responders and non-responders could be distinctly discriminated based on redundant anterior mitral leaflet (AML), increased posterior mitral leaflet (PML) projection, and the AML/PML projection ratio. Notably, all non-responders exhibited a ratio < 1, indicating a markedly anterior displacement of the coaptation point.

These findings are consistent with previous reports suggesting that excessive leaflet elongation and anteriorly shifted coaptation displace the MV apparatus into the left ventricular outflow tract (LVOT), thereby perpetuating obstruction even after effective septal thinning [10,26,27,28]. Unlike surgical myectomy, ASA is inherently unable to correct such intrinsic MV abnormalities, which likely accounts for the persistence of obstruction in certain patients.

The observed ASA success rate in our cohort is comparable to that reported in earlier studies employing similar hemodynamic response criteria. The echocardiographic endpoints used here are in line with international protocolos which are also adopted by high-volume reference centers [15,18,21]. Nevertheless, our study provides novel insights by identifying distinct MV morphological features that appear to predispose to suboptimal ASA outcomes.

Patients with limited hemodynamic response exhibited a constellation of MV abnormalities, most notably a significantly anteriorly displaced coaptation point (AML/PML projection ratio < 1), increased PML projection, and pronounced AML redundancy. These parameters are interrelated and tend to change concordantly, which may explain the lack of statistical independence in multivariate regression analyses due to collinearity. This collinearity, compounded by the relatively small cohort size, further justified our decision not to apply a multivariate model.

A severely anteriorly displaced coaptation point likely contributes to persistent LVOT obstruction after ASA. This can be explained by the fact that ASA primarily targets the basal interventricular septum (IVS), inducing localized akinesia or hypokinesia to reduce septal thickness and contractility, without directly addressing the dynamic role of the MV apparatus in LVOT narrowing. In cases of excessive leaflet elongation, particularly redundant AML and increased PML projection, the MV contributes significantly to residual obstruction via persistent systolic anterior motion (SAM), despite effective septal remodeling. This highlights a key limitation of ASA as a stand-alone therapy, since MV abnormalities represent an independent mechanism of obstruction that may only be adequately corrected by surgical intervention.

Moreover, anterior displacement of the coaptation point and related leaflet abnormalities may not only reflect intrinsic mitral pathology but also broader remodeling of the left ventricle (LV), including changes in ventricular geometry and subvalvular configuration that sustain obstruction independently of septal hypertrophy. Interestingly, ASA outcomes in our cohort were not significantly associated with MV annulus size or absolute AML/PML length. This suggests that relative spatial relationships (e.g., projection ratios, coaptation displacement) and their interaction with adjacent structures such as the LVOT are more relevant predictors of procedural success. These complex interrelations could not be adequately assessed in the present study, given the reliance on two-dimensional echocardiography, which is limited in geometric precision.

In a subset of patients, we performed a pilot analysis using three-dimensional (3D) transesophageal echocardiography (TEE) with GE MVQ software (ECHOPAC version 203) to quantify the mitral–aortic angle. Patients with suboptimal ASA response had a significantly more acute angle, which was inversely correlated with LVOT gradient reduction. These findings align with prior studies [26] and underscore the potential value of advanced 3D imaging parameters in refining patient selection for ASA. Also, contrast 3D echocardiography may be used during the ASA procedure to guide the septal branch selection. Multiple myocardial areas can be assessed simultaneously to exclude contrast spillover, and the targeted myocardial area can be visualized simultaneously in multiple planes, which may improve its demarcation. The 3D technique is currently less described, though for alcohol septal ablation intraprocedural guidance, as compared to other interventions, like transcatheter mitral valve and/or tricuspid valve interventions, left atrial appendage closure, etc. [27,28]. Our results are also concordant with evidence implicating mitral leaflet elongation as a distinctive feature of sarcomeric hypertrophic cardiomyopathy (HCM) compared with phenocopies [29]. For example, Venieri et al. [10] used 3D TEE to demonstrate unique MV features in obstructive HCM, including a more acute aorto-mitral angle, increased annular height, elevated AML-to-LVOT diameter ratio, and anterior displacement of the coaptation line. Notably, annular height correlated with non-sustained ventricular tachycardia, linking MV morphology not only to mechanical obstruction but also to arrhythmic risk. By contrast, other investigations have focused primarily on leaflet length, often reporting inconsistent associations with obstruction severity, which underscores the importance of incorporating additional parameters. Carvalho et al. [30], in a large surgical cohort of 564 patients, found AML elongation in obstructive HCM but did not observe a consistent correlation with LVOT gradient or post-myectomy improvement. Such discrepancies reflect the multifactorial nature of obstruction and the limitations of relying on isolated measurements.

Current intraoperative guidelines recommend surgical MV modification (e.g., AML plication or partial resection of 2–5 mm) when AML length exceeds 16 mm/m^2^ (≈32 mm in absolute terms) or when redundancy is excessive [23,31]. These surgical strategies aim to mitigate SAM and optimize LVOT hemodynamics by reducing AML flexibility and length. Supporting this, Hanein et al. [32] identified AML length as the strongest independent predictor of LVOT obstruction, outperforming conventional metrics such as septal thickness, age, or blood pressure. Similarly, Nara et al. [33] showed that the AML-to-LV end-systolic diameter ratio was independently associated with LVOT gradient, suggesting that leaflet geometry relative to chamber size is more predictive than absolute leaflet length.

Complementary findings have been reported by Song et al. [34], who demonstrated that AML length, spiral LV hypertrophy, and papillary muscle position are independent predictors of obstruction, emphasizing the integral role of MV–subvalvular interactions. In comparison, our study integrated both structural and functional MV parameters—including AML redundancy, projection ratios, and spatial relations to the annulus—providing a more comprehensive appraisal of the MV’s role in ASA response.

Although sarcomeric mutations may directly influence MV morphology, LV remodeling appears to exert a significant impact on leaflet elongation and geometry. Groarke et al. [8] demonstrated associations between sarcomeric mutations, AML elongation, and anterior papillary muscle displacement even in subclinical HCM. Conversely, Chung et al. [35] reported stronger correlations between AML length, LV hypertrophy, and chamber dimensions than with genetic status, suggesting that mechanical stress and secondary remodeling processes may drive MV changes.

Genotype–phenotype interactions have also been highlighted by Guo et al. [36], who found that MYH7 mutations were more frequently associated with elongated leaflets than MYBPC3 mutations. Such data suggest that MV morphology may eventually serve as a non-invasive surrogate marker for genotype, aiding in early stratification of HOCM patients. Despite growing evidence linking MV anatomy to LVOT obstruction, only limited studies have examined its predictive value for ASA success. By contrast, several reports have emphasized LV morphology. Lu et al. [21], for instance, demonstrated that basal anteroseptal plus anterior wall thickness > 50.9 mm predicted ASA success with high sensitivity and specificity. In our cohort, none of the patients reached these thresholds, further underscoring the distinct mechanistic profile captured by MV parameters.

This investigation has several important limitations. First, it was a retrospective, single-center study with a relatively small sample size, which inherently reduces statistical power and limits the generalizability of the findings. Although logistic regression confirmed associations in univariate models, we refrained from performing multivariate analyses because of the small cohort size and high collinearity among MV parameters, which would have yielded unreliable results. Consequently, our findings should be interpreted as exploratory and hypothesis-generating rather than definitive, and require validation in larger, prospective, multicenter cohorts.

Second, the echocardiographic analysis relied primarily on two-dimensional (2D) imaging, which is subject to geometric inaccuracies and highly dependent on acquisition technique. Quantification of leaflet length, projection, and coaptation point displacement requires meticulous image alignment to avoid off-axis measurements. While these methodological constraints are well recognized, the widespread availability and routine clinical use of 2D echocardiography make it a valuable and practical tool in daily practice. Nevertheless, the lack of comprehensive three-dimensional (3D) echocardiographic assessment limited our ability to evaluate advanced geometric parameters, including annular dynamics, leaflet curvature, and tenting area, which may provide additional mechanistic insights. Also, a 3D echocardiographic assessment of the LVOT area before and after ASA would have provided supplemental comparative information for the two groups of patients. The LVOT cross-sectional shape is usually not perfectly circular, and this could be an important source of error when measured with 2D echocardiography, in which the circle area formula is used for calculation (assuming a circular LVOT geometry). This formula involves squaring the measured LVOT radius, and, thus, any error would be significantly amplified [37].

Third, the small number of non-responders precluded the establishment of reliable cutoff values for MV parameters predictive of ASA success. Defining such thresholds will require prospective validation in larger cohorts, ideally with standardized 3D echocardiographic or multimodality imaging protocols.

Finally, this study did not incorporate systematic genetic testing, which may have helped disentangle the relative contributions of sarcomeric mutations and LV remodeling to MV morphology. Given the emerging evidence of genotype–phenotype interactions in shaping MV geometry, integration of imaging, hemodynamic, and genetic data in future research may enable more precise patient stratification and therapeutic tailoring.

## 5. Conclusions

Mitral valve morphological features—particularly redundant AML, greater PML projection, and anterior displacement of the coaptation point—can serve as important predictors for suboptimal ASA outcomes in univariate analysis. These data emphasize the need for comprehensive MV imaging, ideally incorporating 3D modalities, in pre-procedural assessment. Integrating MV morphology into current selection algorithms may refine ASA patient selection and improve long-term success rates.

## Figures and Tables

**Table 1:** Clinical profile of the two groups of patients.

	Responders’ Group *n* = 32	Non-Responders’ Group *n* = 6	*p* Value
Age, years	55.81 ± 13.24	57.5 ± 8.8	0.76
Male sex, *n* (%)	12 (37.5%)	4 (66.66%)	0.21
DM, *n* (%)	6 (18.75%)	1 (16.66%)	1
Obesity, *n* (%)	7 (21.87%)	2 (33.33%)	0.61
CKD, *n* (%)	4 (12.5%)	1 (16.66%)	1
HTN, *n* (%)	23 (71.87%)	3 (50%)	0.35
Atrial fibrillation, *n* (%)	2 (6.25%)	1 (16.66%)	0.41
NYHA class, *n* (%)			0.7
II	11 (34.37%)	3 (50%)	
III	20 (62.5%)	3 (50%)	
IV	1 (3.12%)	0 (0%)	
NYHA class	2.68 ± 0.53	2.5 ± 0.54	0.43

*n*—number, DM—diabetes mellitus, CKD—chronic kidney disease, HTN—arterial hypertension, and NYHA—New York Heart Association.

**Table 2:** General echocardiographic features of the two groups of patients.

	Responders’ Group *n* = 32	Non-Responders’ Group *n* = 6	*p* Value
Basal IVS, mm	23.4 ± 3.98	24.5 ± 4.13	0.44
Basal anterior wall, mm	21 ± 2.47	21.66 ± 2.5	0.54
LVEDD, mm	38.56 ± 12.31	44.5 ± 3.39	0.25
LVEDV, mL	98.87 ± 24.58	110.33 ± 32.95	0.32
LVEDVi, mL/m^2^	52.66 ± 12.42	55.22 ± 11.95	0.64
LVEF, %	65.56 ± 7.2	69 ± 4.04	0.26
LAV, mL	116.84 ± 40.08	111.66 ± 29.66	0.76
LAVi, mL/m^2^	62.63 ± 21.62	56.70 ± 13.56	0.52
Maximum baseline LVOT PG, mmHg	100.25 ± 27.75	106.5 ± 21.57	0.6

*n*—number; mm—millimeters; IVS—interventricular septum; LVEDD—left ventricular end-diastolic diameter; LVEDV—left ventricular end-diastolic volume; mL—milliliter; LVEDVi—indexed left ventricular end-diastolic volume; mL/m^2^—milliliter/square meter of body surface; LAV—left atrial volume; LAVi—indexed left atrial volume; LVOT PG—left ventricular outflow tract pressure gradient.

**Table 3:** Mitral valve echocardiographic parameter differences.

	Responders’ Group *n* = 32	Non-Responders’ Group *n* = 6	*p* Value
Mitral regurgitation, *n* (%)			0.15
Mild	21 (65.62%)	6 (100%)	
Moderate	11 (34.37%)	0 (0%)	
Mitral annulus diastole, mm	31.06 ± 3.6	33.66 ± 7.78	0.19
Mitral annulus systole, mm	30.56 ± 3.83	33.5 ± 7.71	0.15
AML length, mm	29.21 ± 3.42	29.83 ± 5.51	0.71
Redundant AML, mm	9.96 ± 1.99	13.16 ± 1.72	<0.001 *
PML lenght, mm	17.62 ± 4.19	21.5 ± 4.84	0.04 *
AML projection, mm	0.55 ± 0.09	0.44 ± 0.05	0.007 *
PML projection, mm	13.65 ± 3.8	18.5 ± 3.78	0.006 *
AML/PML projection	1.34 ± 0.45	0.8 ± 0.15	0.007 *

*n*—number; mm—millimeters; AML—anterior mitral leaflet; PML—posterior mitral leaflet; *—statistically significant.

**Table 4:** Correlation matrix proving a statistically significant correlation between the percentage of LVOT PG decrease and the redundant AML, PML projection, and the anterior displacement of the coaptation point (AML/PML projection ratio). See text for details. LVOT PG—left ventricular outflow tract pressure gradient, IVS—interventricular septum, LVAW—left ventricular anterior wall, AML—anterior mitral leaflet, PML—posterior mitral leaflet, Red. AML—redundant anterior mitral leaflet.

	LVOT PG Decrease	IVS	LVAW	AML	PML	Red. AML	AML Projection	PML Projection	AML Projection/PML Projection
**LVOT PG decrease**	—								
**IVS**	0.201	—							
**LVAW**	0.064	0.323 *	—						
**AML**	0.093	0.173	−0.199	—					
**PML**	0.249	0.061	0.057	0.520 ***	—				
**Red. AML**	0.460 **	0.196	0.117	0.483 **	0.561 ***	—			
**AML projection**	−0.258	−0.106	−0.147	0.515 ***	0.062	0.061	—		
**PML projection**	0.360 *	0.109	0.056	0.342 *	0.787 ***	0.573 ***	−0.202	—	
**AML projection/PML projection**	−0.398 *	−0.104	−0.148	0.114	−0.479 **	−0.283	0.665 ***	−0.796 ***	—

* *p* < 0.05, ** *p* < 0.01, *** *p* < 0.001.

**Table 5:** Univariate logistic regression model for the impact of the redundant AML length, PML projection and AML projection/PML projection ratio on procedural success.

Predictor	Estimate	SE	Z	*p* Value
Intercept (Redundant AML model)	10.401	3.669	2.83	0.005
Redundant AML length (mm)	−0.755	0.295	−2.56	0.010
Intercept (PML projection model)	5.922	2.081	2.85	0.004
PML projection length (mm)	−0.270	0.122	−2.22	0.027
Intercept (AML/PML ratio model)	−3.40	1.94	−1.75	0.079
AML/PML projection ratio	4.87	2.00	2.43	0.015

Estimate—log odds of procedural success.

## Data Availability

Data are contained within this article.
